# Sensory Stimulation as a Means of Sustained Enhancement of Well-Being in Leopard Geckos, *Eublepharis macularius* (Eublepharidae, Squamata)

**DOI:** 10.3390/ani13233595

**Published:** 2023-11-21

**Authors:** Frank Krönke, Lisa Xu

**Affiliations:** 1 Auffangstation für Reptilien e.V. München, Rescue Center for Reptiles, Kaulbachstrasse 37, 80539 München, Germany; 2 Statistical Consulting Unit, StaBLab, Department of Statistics, Ludwig-Maximilians-Universität, Ludwigstrasse 33, 80539 München, Germany

**Keywords:** behavioural preferences, enrichment evaluation, *Eublepharis*, evidence-based husbandry, reptiles, well-being, welfare

## Abstract

**Simple Summary:**

The leopard gecko is probably the most common lizard that is kept in terrariums worldwide. In private keeping, its popularity has remained unbroken for decades, and it is also used in science to research a wide variety of questions. The aim of this study was to improve the husbandry by simple means in order to increase the well-being of the animals in the terrarium. For this purpose, the animals were fed very small insects instead of large ones. The intention behind this was that this would create much more varied and lasting stimuli that would encourage the leopard geckos to show more behaviour on the one hand and more varied behaviour on the other. Both are considered signs of increased well-being. The results confirmed this expectation and showed that even after 11 months of continuous feeding with small insects, the behavioural frequencies had almost doubled. The behavioural diversity had increased less, but both results could show that the method used had clearly achieved its goal. This study has thus made an important contribution to improving the husbandry of leopard geckos by showing that a small intervention, which is possible for every keeper and does not require more time or money, can achieve a significant increase in well-being and, thus, species-appropriate husbandry.

**Abstract:**

Although the private keeping of reptiles has boomed in most western countries since the millennium, studies dealing with the recognition and promotion of welfare in these reptiles seem to represent a blind spot of scientific attention. The vast majority of studies from the field of animal welfare science still concern mammals and birds. The leopard gecko is probably the most common lizard that is kept in domestic terrariums worldwide. Due to its characteristic as an ecological generalist, it is easy to keep and breed, and it is considered a good “starter reptile” for beginners as it “condones” husbandry mistakes, even for extended periods. However, being a mass species is not a second-class classification. They, too, have an equal claim to good well-being as all animals in human care. The aim of the study was to test the hypothesis of whether an increase in stimulus density leads to an increase in activity and behavioural diversity and, thus, an increase in welfare. For this purpose, 18 leopard geckos were fed insects that were ≤1 cm in size, and both the quantity and quality of behaviour was documented and analysed in the pre-intervention, intervention and post-intervention stages. In addition, it was of interest whether behavioural indicators could be identified that indicate a state of positive well-being. The results showed that this type of enrichment led to a quantitative doubling of the activity levels from the baseline (total of 12,519 behavioural elements) to the intervention (total of 25,366 behavioural elements). And even 11 months after the introduction of small insect feeding (post-intervention total of 23,267 behavioural elements), the activity level was still significantly increased. The behavioural diversity, as the absolute number of behavioural categories across all 18 leopard geckos, also increased, although less than the behavioural intensity, between the baseline (5507 behavioural categories) and intervention (6451 behavioural categories) and between the baseline and post-intervention (6079 behavioural categories). The results clearly show that feeding small insects to leopard geckos is a very efficient tool to increase the welfare of leopard geckos. Attractively, this feeding regime can be implemented by any leopard gecko keeper without significant additional cost or time, and therefore, these methods have a potentially high impact.

## 1. Introduction

“Animal welfare is increasingly recognised as a high priority for modern zoos and aquariums” [1]. Statements like this reflect the current debate, cf., [2,3,4], about welfare. Looking at this statement in more detail, it quickly becomes apparent that the majority of studies and interventions concern mammals and birds, while information on reptiles is rare [5,6,7,8,9,10,11]. It is astonishing that the private keeping of reptiles seems to be an almost blind spot of scientific interest from the perspective of animal welfare science. In the 1990s, the interest in private reptile keeping increased—parallel to the development and spread of the internet. This trend grew into a real boom from around the turn of the millennium onwards [9,12,13,14,15,16]. Currently, there are millions of privately kept reptiles in Europe, the USA and parts of Asia [16,17].

There is no universal definition of animal welfare because it is not only a scientific concept, but also reflects ethical standpoints and different practical views [2,4,18]. Animal well-being implies physical, behavioural and psychological aspects, as well as their complex interactions. A good state of welfare means being healthy, well nourished, feeling comfortable, having a sense of security, being able to express a range of innate/normal behaviours and the absence of suffering, fear, pain or persistent distress. Welfare is a long-term state that comprises the sum of experiences of an animal and its derived affective state [3,4,19,20]. For these states to be realised, coping abilities—physiological, behavioural, or cognitive—are important factors, too, such as being allowed to choose and the ability/self-efficacy to reach desirable outcomes [2,21,22,23,24,25,26] under conditions of their substitute habitat in the terrarium. In other words, “[...] an animal’s behaviour is interpreted in terms of what it is intending to achieve, its active and positive engagement in goal-directed behaviours, the rewards it may experience when goals are achieved, and frustration when thwarted, and the wide range of pleasurable experiences that animal may have. These experiences may now be suggested to include feelings of satiety, appetitive and consumatory satisfaction, reward, goal-directed engagement, curiosity, vitality, […], calmness, contentment, […], and feelings of security” [18].

The concept of “behavioural preferences” [27,28] or “highly motivated behaviour” [29] plays an important role in assessing the welfare state of an animal. These behaviours show what the animal wants or does not want to do, from which it can be deduced that positive motivations to act are associated with positive mental states and welfare, and avoidance behaviour or frustration are associated with negative mental states and negative welfare. The subjective, affective state of an animal is therefore at the centre of the consideration [19,20,27,28,29,30,31]. Consequently, behavioural indicators can also be derived from this, which can be used to assess the welfare state [30].

A fundamental problem in animal husbandry in general, as well as in terrarium husbandry in particular, is the lack of stimuli and the resulting lack of behavioural and cognitive opportunities, which has a negative impact on animal welfare [7,20,22,26,30,32]. Depending on the needs and abilities of an animal [33,34], stimuli can be offered that specifically create behavioural opportunities to promote behavioural preferences and thus well-being [20,22,30,32,35], which is referred to as environmental enrichment. It is a means to reach a higher degree of animal welfare as well as an essential element of good husbandry, cf., [3,7,10,36,37]. It offers the animal the opportunity to use and train its behavioural and cognitive skills and abilities, while, at the same time, exercising control, i.e., changing an immediate environment according to its needs [21,22,24,25,26]. Enrichment interventions should always include two timelines: a short-term one, where the focus is on whether the stimulus provided is attractive to the animal and is accepted, and a long-term one, within which the set goals are to be achieved, e.g., increasing activity or reducing conditions that are associated with reduced well-being such as boredom [5,12,22,23,24,25,37,38,39,40].

Although the majority of enrichment studies have been conducted with mammals, there are some that clearly show that this approach works equally well with reptiles. For example, it has been shown that corn snakes (*Pantherophis guttatus*) that received greater structural diversity in their housing conditions as enrichment compared to the control group performed more exploratory behaviour, showed more sophisticated cognitive performance and were more interested in new stimuli [41]. The study by Almli et al. [42] points in the same direction, showing that black rat snakes (*Pantherophis obsoletus*), which also received a significantly more diversified enrichment in their terrarium compared to the control group, showed superior abilities and shorter latencies in problem solving tasks and habituated more quickly to new situations or stimulus objects. Another study with *Pantherophis guttatus* showed that an increase in environmental complexity increased the behavioural intensity and welfare of these animals [43]. For aquatic turtles (*Pseudemys* sp. and *Trachemys* sp.), it was shown that the provision of coloured stimuli triggered significant interest and exploration of these objects, thereby significantly reducing behaviours of low well-being (escape behaviour) [44,45]. In green turtles (*Chelonia mydas*), it was shown that animals that received plastic objects as enrichment items for exploration bit each other less [46]. Bryant et al. [47] offered fly river turtles (*Carettochelys insculpta*) a plastic object filled with food as a food enrichment item. As a result, the turtles spent about 40 min acquiring food as opposed to 5–6 min under standard feeding conditions. When North American box turtles’ (*Terrapene carolina*) housing was enriched by providing them with natural bottom substrate and hiding places, they showed significantly less escape behaviours and thus higher welfare [48]. Londoño et al. [49] demonstrated that chemosensory enrichment for Catalonian wall lizards (*Podarcis liolepis*) resulted in a significant decrease in abnormal behaviours and an increase in normal behaviour and well-being. Leopard geckos showed increased activity, cognitive performance and well-being under conditions of increased housing complexity [50,51].

While enrichment for reptiles is used in some European zoos, Bartolomé et al. [11] highlight in their recent study both the need to consider a wider array of enrichment types, as well as to increase the number of reptile species studied, and to validate different methods of welfare assessment. The factors that led zoos to enrich a specific reptile species were increased activity and food intake (i.e., ease of enrichment), a large body size, an advanced age and charisma as a zoo animal. From these statements, the necessity of the present study can be derived both directly and indirectly, in that leopard geckos neither play roles as zoo animals nor are they particularly charismatic, but due to their enormous worldwide popularity, they receive a great deal of attention in private husbandry.

Food enrichment plays an important role in reptile husbandry [11] because these animals show comparatively little social behaviours and only very rarely show play behaviours. Food satisfies a strong basic need and generates a wide variety of behaviours because it appeals to different senses [38,52,53,54,55]. Through the necessary additional effort of food acquisition, the time budget is shifted more in the direction of natural living conditions, boredom caused by a lack of stimuli is reduced and physical and cognitive challenges are created, which increases welfare [56,57]. Besides mere foraging, the stimulation of exploratory behaviour is of particular importance for all captive animals, especially for foragers and predators adapted to natural habitats characterised by the high unpredictability of their food organisms in time and space, because investigative exploration provides important information on various environmental aspects such as predator avoidance and knowledge about the occurrence of food or water or the locations of shelters [22,39,57,58].

Leopard geckos (*Eublepharis macularius*) are probably the most common lizard species that are kept in terrariums worldwide [59,60]. The first geckos probably arrived in Europe as early as the late 1950s [61]. They are kept as pets and are also used as a model organism in a wide variety of scientific research, e.g., [62,63,64,65,66,67,68,69]. They are considered easy to keep and breed. This is due to their wide ecological amplitude, meaning they forgive the keepers who persistently provide sub-optimal husbandry. But surviving is not thriving.

*Eublepharis macularius* (Eublepharidae) is distributed from south-east Iran [70], over Pakistan, north-west India [71] and southern Nepal [72] from the plains up to an elevation of 3200 m [73], which includes a variety of different climatic zones [74]. They prefer stony or rocky areas as well as mudflats with little vegetation, and in some regions, they also prefer open dry forests. They are nocturnal, and in the terrarium, they are also crepuscular. Their preferred hiding places are under stones, in holes in the ground and sometimes under loose bark or in rock walls. They often live in loose aggregations, probably depending on resource availability [71,75]. Under ordinary (small) terrarium conditions, males do not get along with each other and should therefore be kept individually [76,77]. Their diets consist of beetles, crickets, spiders, scorpions and other arthropods, which are not evenly distributed throughout the year. In some areas of its habitats, the leopard gecko hibernates from October/November to February [71,75]. Agrawal et al. [69], by means of genetic samples, estimated that all captive-bred leopard geckos originated from Pakistan. Leopard geckos can reach an astonishingly high age in captivity under good husbandry conditions: the oldest documented animal died at the age of 46 in 2022 (E. Laue pers. comm.).

All animals in human care must have all their needs met and their welfare maximised [3,36,78,79,80,81,82,83,84]. This should be a basic requirement in any animal husbandry practice. However, in order to meet this requirement, it is necessary to evaluate husbandry practices in order to assess the resulting impact on welfare [7,10,11,12,21,27]. Such data would make it easier for both private reptile keepers and official veterinarians to assess husbandry conditions.

The focus of this study was the creation of evidence-based reptile husbandry regimes, cf., [5,12,19,43,55,85], to improve the living conditions and welfare of leopard geckos in both private and scientific contexts [8,43,86,87]. Therefore, this study tested the hypothesis of whether increasing the stimulus density in the terrarium can increase the expression of behavioural preferences and thus well-being. For this purpose, 18 leopard geckos were fed insects ≤1 cm in size, and both the quantity and quality of behaviour was documented and analysed in the pre-intervention, intervention and post-intervention stages. In addition, it was of interest whether behavioural indicators could be identified that indicate a state of positive well-being.

## 2. Materials and Methods

### 2.1. Experimental Design

**Figure 1:**
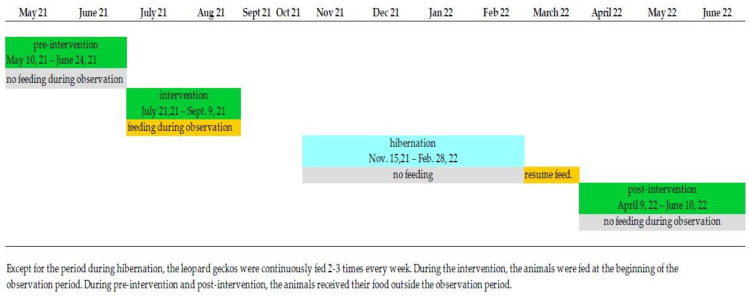
Timeline observation and feeding scheme.

In order to be able to determine whether increasing the stimulus density with the help of small insects causes a change in behaviour in leopard geckos, three data sets were collected: pre-intervention (10 May 2021 to 24 June 2021), intervention (21 July 2021 to 9 September 2021) and post-intervention (9 April 2022 to 10 June 2022). see Figure 1.

The design of the focal sampling was as follows: in each set, each gecko was observed in its own terrarium for 30 min a day for 14 days during its evening peak activity time between 6 pm and 11 pm. The daily observation time was divided into 6 × 5 min. Since it was not possible to observe all 18 geckos on the same evening, they were clumped into three observational sets of 6 animals. For each group, there were 36 observation units per evening, which were randomly distributed over three hours of observation time. In total, there were 84 observation units/set/animal, resulting in 1512 observation units/all animals, or 126 h of observation time/all geckos/set.

The pre-intervention set was the baseline survey and served as a control, since we recorded what kind of behaviours occurred and how often the animals showed the behaviours without any stimulus. This approach seems justified because “When two groups of animals of the same species are to be compared—for example in relation to their activity budgets with and without the presence of an enrichment feeder—the only way to ensure that the groups have identical characteristics is to use the same subjects in each group. As each individual is in both groups, subject variables will balance out equally, so this is the most effective method of control” ([88], p. 30).

During the intervention survey, an amount of approximately 1 g of insects was added to each terrarium each evening before the first observation. As the terrariums were naturally furnished, the insects had a variety of hiding places, which they immediately sought out and subsequently repeatedly left for short periods. This created unpredictable odours, acoustics and visual stimuli on the entire surface of the terrarium. No feeding was carried out during the post-intervention survey, but outside of the fortnightly observation period, small insect feeding was maintained since its introduction. For the whole study, 4536 observation units were conducted, which means there was 378 h of data collection. All observations and recordings were conducted by the lead author (FK).

### 2.2. Behavioural Observations

The behaviour of leopard geckos is not very complex, and it is characterised by slow movements interrupted by numerous pauses. Therefore, recording their behaviour with pen and paper was suitable. The observations took place in front of the closed terrariums, after the lighting had been switched off by a timer at about 6 pm, at a distance of about 1 m. Every predefined behaviour documented in the ethogram was recorded. Only the occurrence of a behaviour within the observation interval was counted, and not its length. If behaviour A was replaced by behaviour B and then behaviour A was shown again, then behaviour A was counted twice, and so on. After each observation unit, the terrarium was opened, and the geckos’ body temperatures were measured in the middle of the back with an infrared thermometer. Since all geckos were used to living under terrarium conditions and people handling them, they did not seem disturbed by this observational setting.

### 2.3. Ethogram

In a preliminary investigation, the leopard geckos were observed in their terrariums over a period of about three weeks (=approx. 40 h) during their main activity time, and all behavioural elements that appeared relevant were recorded and described. Only when no more new behaviours were shown was the preliminary investigation terminated. At the same time, we tested how far away the observer had to be from the terrarium so that the animals did not react to him, how indirect lighting was optimally designed or how long the activity span of the animals lasted in the evening. In the course of the study, it became apparent that various behavioural elements were regularly related. These were grouped into different superordinate categories in the course of the data evaluation (see Table 1).

### 2.4. Study Animals

Eighteen adult leopard geckos (see Table 2) were studied in this study, including ten females and eight males. The weights of the females were between 38 and 72 g, and those of the males were between 40.5 and 87 g. No information can be given about the exact age. All leopard geckos had hatched and grown up under terrarium conditions and were therefore used to contact with humans as well as the specific environmental conditions (light and temperature). Only the animals that had high to excellent physical and behavioural health were included in this study. Health assessment was conducted via an external assessment by a specialist veterinarian for reptiles. Furthermore, several faecal samples/animal were examined for parasites, and most geckos were treated with medication against intestinal parasites before the start of the study. In the subsequent pre-study, the animals were assessed for behavioural anomalies.

### 2.5. Housing and Husbandry

**Figure 2:**
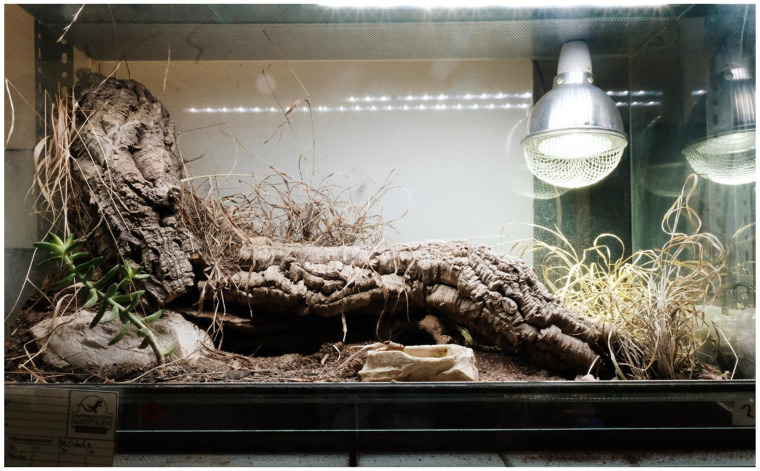
Example of a study terrarium: several hiding places, LED lights, heat lamp, natural furnishings and opportunities to climb and burrow.

**Figure 3:**
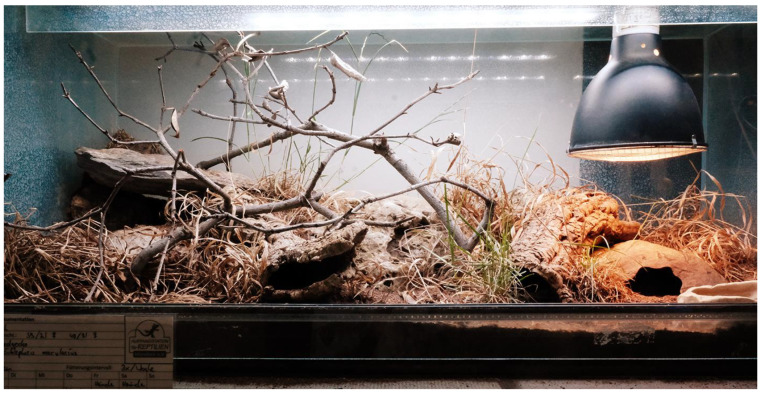
Example of a study terrarium: several hiding places, LED lights, heat lamp, natural furnishings, opportunities to climb and burrow.

**Figure 4:**
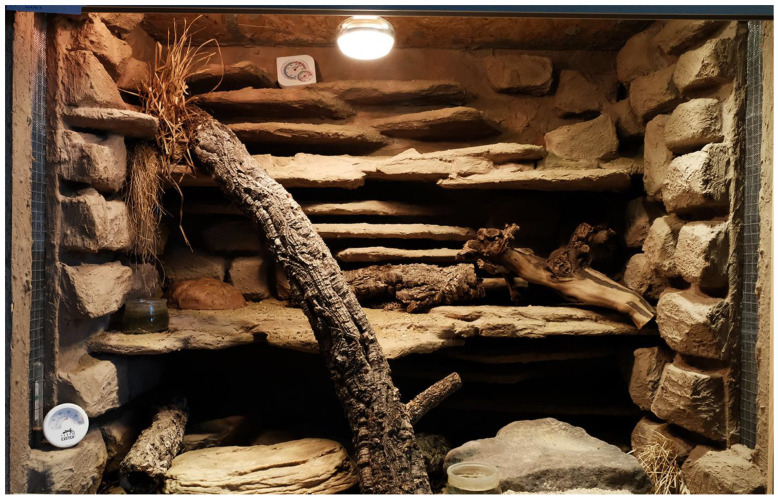
Example of a study terrarium: natural furnishing, opportunities to climb, opportunities for thermoregulation, multitude of hiding places, ground layer with several levels, heat lamp.

**Figure 5:**
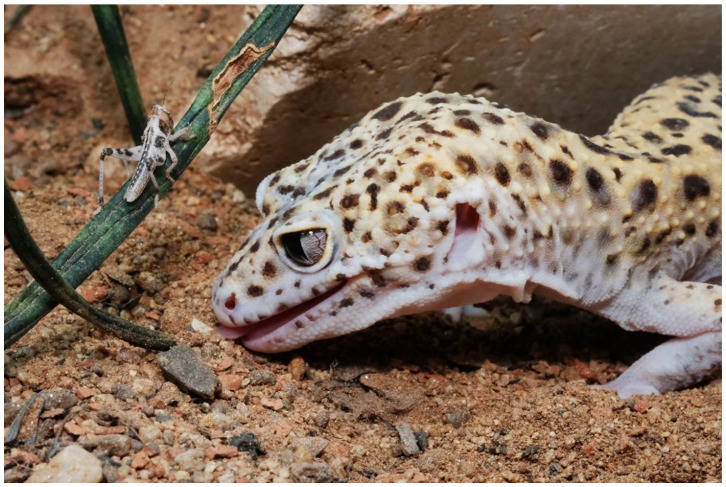
Leopard gecko taking vomeronasal information.

**Figure 6:**
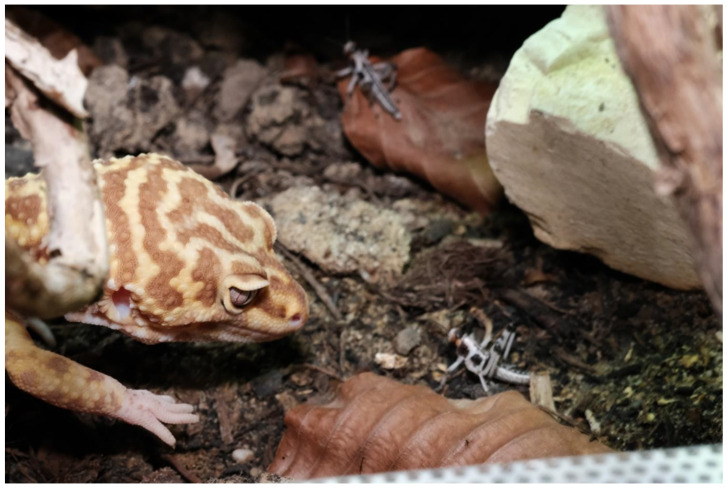
Leopard gecko is interested in desert locusts.

The leopard geckos were housed in the same terrarium during the whole study. The floor space for individually housed geckos was between 2500 and 4000 square centimetres each, and for group-housed geckos, the floor space was between 4000 and 21,000 square centimetres (see Table 2). Every terrarium was provided with a 10 cm layer of loamy sand, several stones or stone flags, roots or branches and several small cork tubes and a water bowl. Thus, every individual had at least three different hiding places available. Some terrariums additionally had rear and side walls constructed with artificial clefts to increase the useable space, create additional hiding places and to facilitate thermoregulation (see Figure 2, Figure 3 and Figure 4). All geckos were previously fed mainly with adult insects (house crickets and locusts). Every terrarium had a heat lamp. The surface temperatures within each terrarium were kept between approximately 25 and 45 °C (temperature gradient) during daytime. The air temperature at night was kept between 17 and 26 °C (depending on the season). Every terrarium room, except for one, had at least one window and access to daylight. Every other day, the terrariums were sprayed with water in order to raise the humidity and to keep some parts of the substrate humid, faeces was removed and the water bowl was cleaned and refilled. From mid-October to early November, all leopard geckos became sluggish, and the study was interrupted from 15 November 2021 to 28 February 2022, as all animals were hibernating. The hibernation temperatures ranged from 10 to 16 °C. On 15 March 2022, feeding was resumed. Feeding took place 2–3 x/week; alternately, house crickets, field crickets, desert locust or flightless houseflies were fed to the geckos. All three observation groups received the different types of feeding insects in the same frequency. Within a group, only one type of insect was offered per feeding. Overall, all insect species were offered equally often. The same insect species was offered on a maximum of two evenings in a row. Each leopard gecko was offered approx. 5 g of insects per week, dusted with a calcium vitamin preparation (Korvimin for reptiles and birds). If there was more than one animal in the terrarium, this amount was multiplied by the respective number of animals. The insects were always put into the terrarium alive and immediately dispersed and hid. Feeding was carried out during the whole observation period, excluding the hibernation period. Only during the intervention study were the geckos fed immediately before data collection, and in all other cases, the geckos were fed outside the observation period. In this way, it was possible to elicit a strong behavioural amplitude that could be used as a benchmark for a high level of well-being (hunting behaviour, feeding and exploration), and this was particularly useful for assessing the long-term effect of small insect feeding, see Figure 5 and Figure 6. As the observation times per leopard gecko (6 × 5 min) were randomly distributed over the total observation time per evening, there should not have been bias in the data.

### 2.6. Sex

As shown in Table 2, males were mostly kept individually, whereas females were always kept in groups of two or, in a big terrarium (21,000 square centimetres with several “floors”), in a group of four. They did not interact much with each other, but often shared the same hiding place. Sometimes, they were attracted by the prey that another gecko had just caught. From time to time, one walked over another. Reactions to this behaviour included stoically ignoring it, avoiding the other or, in some rare cases, snapping at the other animal. These interactions were not counted as behavioural units to maintain comparability between individually kept and group-kept animals, as well as same-sex and opposite-sex groups. Some leopard geckos were kept in male–female couples, which had known each other for several years and were in tune with each other. A male never walked over a female, and if the female walked over a male, the male always ignored it. Behaviour related to these interactions was likewise not counted.

### 2.7. Feeding Insects

Four insect species with different behavioural characteristics were offered as stimuli to the leopard geckos:

House crickets, *Acheta domesticus*; field crickets, *Gryllus assimilis*
  They quickly seek a hiding place, move quickly in intervals, congregate in warm places, spend more time under a cover than in open space, move mostly on the ground and are active during the day and in darkness; field crickets were less active than the house crickets.
Desert locust, *Schistocerca gregaria*
  During the day, they often stay out of the cover, and they hide at dusk and during the night; they move slowly or jump, climb on all kinds of elevated points and gather in warm places.
Housefly, *Musca domestica*
  They are incapable of flying, they do not seek out hiding places, they distribute themselves over all surfaces, are active above all during the day and gather in warm places. Their inability to fly is due to a defect mutation and it is common in the reptile feed market.

### 2.8. Forceps Feeding

In order to have a time comparison of different feeding methods, in a preliminary study, it was measured how long it took a leopard gecko to eat three adult house crickets offered to it with tweezers. This corresponds to about half of the weekly feeding ration. A total of 30 timings took place with the 18 leopard geckos. The starting point was the visual fixation of the insect, and the end point was the swallowing process.

### 2.9. Intrinsic Rhythms

The observation time followed the intrinsic schedule of the animals that became active immediately before or after the terrarium lights switched off at around 6 p.m. Since crepuscular reptiles have an activity maximum in the first half of the night due to dropping temperatures [89], there was no necessity to conduct observations after midnight. This prediction was confirmed by the observational and statistical data, which showed decreasing activity as the time progressed.

Another intrinsic schedule was the decreasing activity of most leopard geckos around mid-October, although neither the temperature nor quality or quantity of the terrarium lightning changed. Two things appeared interesting: 1. Only 14 of 18 geckos became less active; all of these animals had big windows in their terrarium rooms. The enclosures of the remaining four geckos were windowless rooms, and these animals showed no decrease in activity. These observations were in accordance with the descriptions from the literature [90]. 2. About half of the animals were used to a hibernation period of about three months, and the other half were not used to this. Regardless, the decrease in activity was about the same in both groups. This study was therefore interrupted, and all of the leopard geckos had 14 weeks of hibernation, followed by about four weeks of re-acclimatisation.

### 2.10. Technical Equipment

To measure the body temperature, a Testo 831 (Testo SE & Co. KGaA, Lenzkirch, Germany) infrared thermometer with a two-point laser was used. Two 5-Watt LED lamps were used to create an indirect light in the terrarium room, just bright enough to see the details of the body movements.

### 2.11. Statistical Analysis

In order to compare the behaviours of the individual animals over the study, all observed behavioural units were summed up for each animal, survey and behavioural category. That enabled us to create one variable for each animal and behavioural category, indicating the observed number of behavioural units for the baseline, intervention and post-intervention stages. Those numbers of behavioural units were first descriptively examined for each study stage. After data preparation, a paired *t*-test was performed to determine whether there was a significant change in the behaviours between the three individual data collection stages. To investigate the differences in behaviours between the three different food insects, one-way ANOVA was used. In addition, for the examination of the differences between male and female animals, an unpaired *t*-test was performed. In general, all tests were performed with significance level = 0.05.

**Figure 7:**
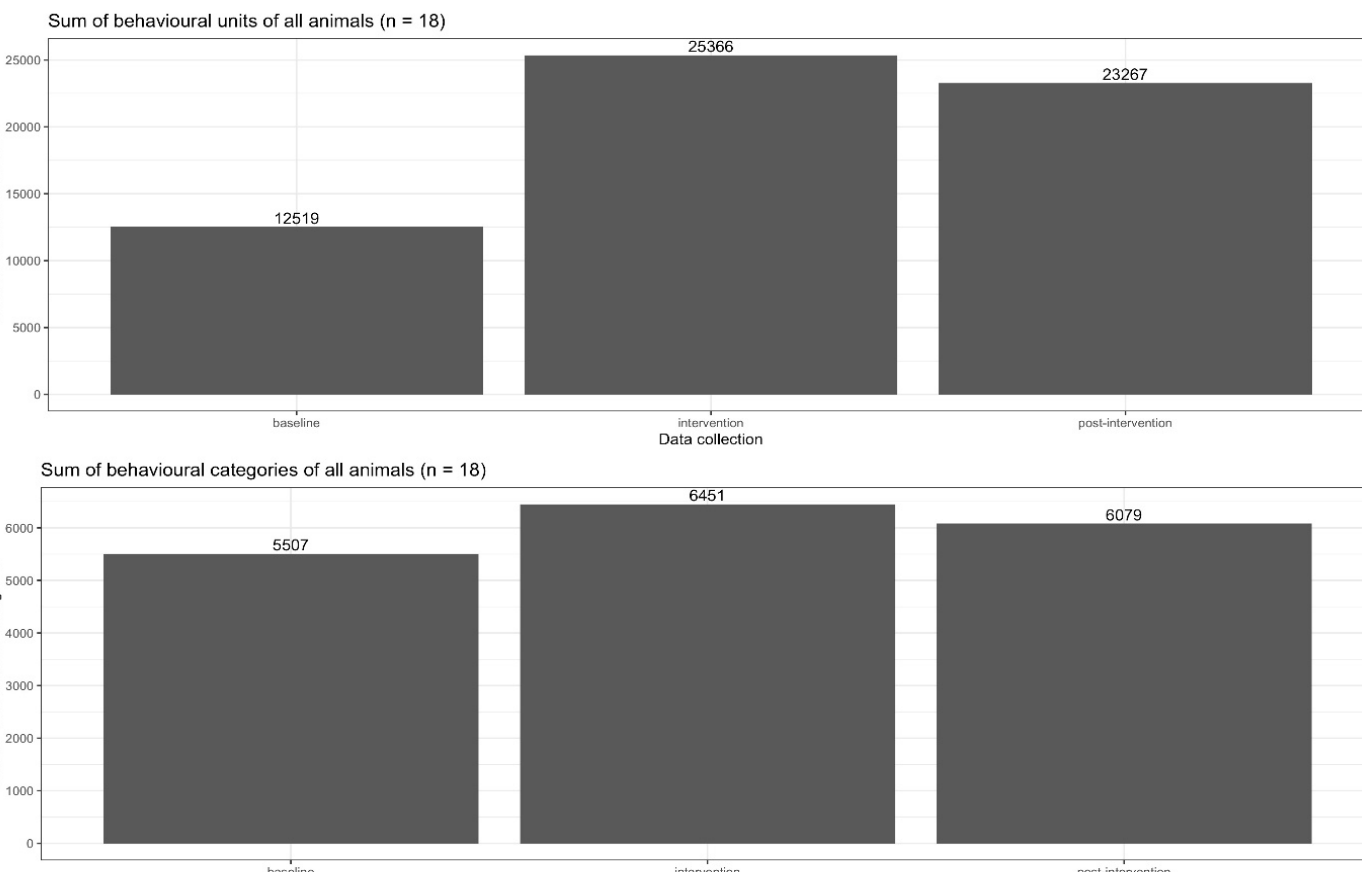
Total sum of behavioural units and total sum of behavioural categories of all animals.

Apart from the number of observed behavioural units, we wanted to assess the diversity of the behavioural categories since behavioural diversity is considered a potential indicator for animal welfare. We therefore calculated the share of performed behavioural categories out of a total of 31 possible ones (see Table 1: Ethogram). For this share, it was only relevant whether the category was observed at least once in each data collection point. The frequency of the observed category is not considered here since we are only interested in the variety of different categories realized. The overall frequency of behavioural categories is presented in Figure 7. All analyses were performed using R (Version 4.1.0).

### 2.12. Qualitative Data

There were observations that appeared to be important but occurred outside the defined observation timeslots. In some cases, this was a rare behaviour that occurred in a different context than the study and therefore was not quantified, but it remains an important cue to assess a certain situation.

## 3. Results

### 3.1. Sensory Stimulation as a Means of Sustained Enhancement of Well-Being

**Figure 8:**
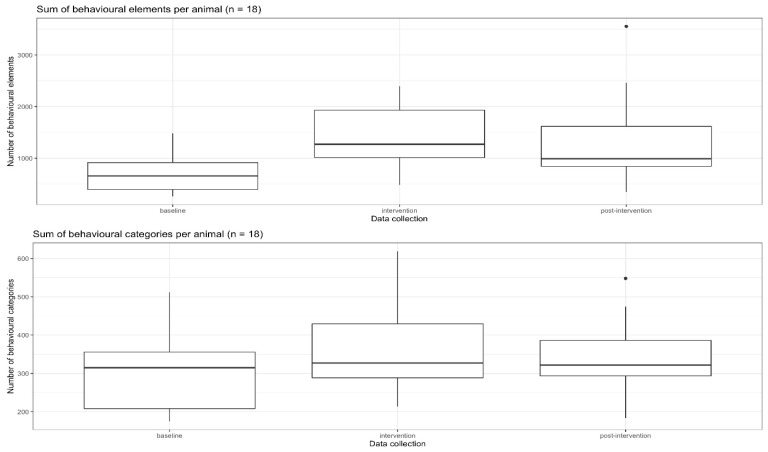
Total sum of behavioural elements per animal.

A comparison of the baseline and intervention data sets showed significant increases in the mean number of behavioural units from 696 to 1409 (*p*-value = 0.001) and between the baseline and post-intervention from 696 to 1293 (*p*-value = 0.001). In total, 12,519 behavioural units were counted in the baseline survey of all 18 leopard geckos, 25,366 behavioural units were counted in the intervention survey and 23,267 behavioural units were counted in the post-intervention survey, 11 months after the start of the intervention (see Figure 7 and Figure 8). The proportion of foraging during the intervention survey was minimal; 4.1% of foraging was related to all activities within this survey.

The effect on behavioural diversity, measured in behavioural categories, was less strong. Considering the absolute numbers of counts of behavioural categories, between the baseline and the intervention sets, there was an increase from 5507 to 6451, and 11 months later, in the post-intervention, there were 6079 behavioural categories realised by all 18 leopard geckos (see Figure 7 and Figure 8). From the baseline to the intervention set, the mean number of behavioural categories/animal increased significantly from 306 to 358 (*p*-value = 0.043). The increase from the baseline to post-intervention was 338 and was not significant (*p*-value = 0.142).

**Figure 9:**
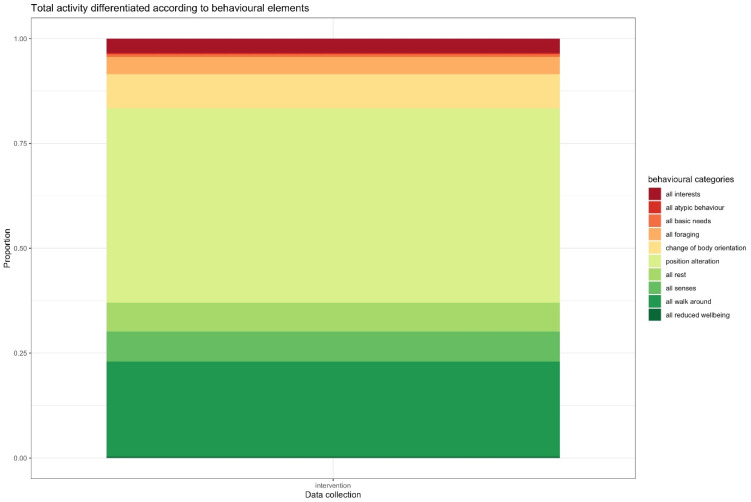
Total activity differentiated according to behavioural elements.

**Figure 10:**
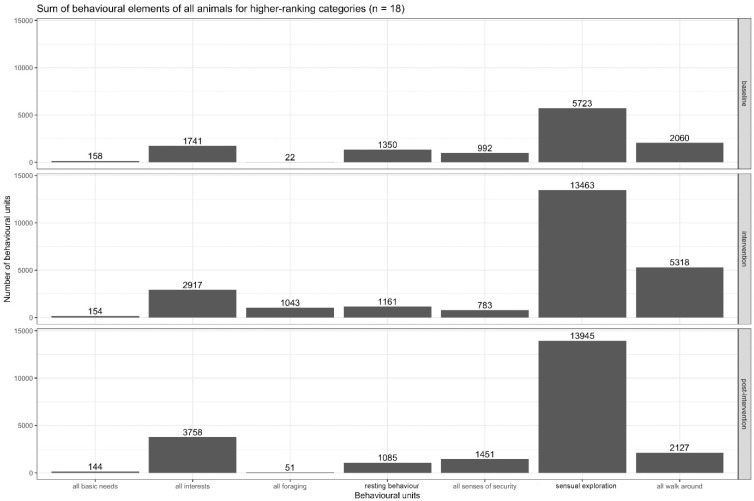
Total sum of behavioural elements of all animals for higher-ranking categories.

If the number of realised behavioural categories out of the 31 possible categories (ethogram) is considered, only a significant increase in behavioural categories between the intervention (0.670) and post-intervention sets (0.578, *p*-value = 0.005) can be observed (see Table 3). The total activity differentiated according to the behavioural elements is visualized in Figure 9. The total sum of behavioural elements of all animals for higher-ranking categories is presented in Figure 10.

### 3.2. Details Related to Co-Variables

#### 3.2.1. Sex

Eight males showed 26,049 behavioural units, whereas ten females realised a total of 35,103 behavioural units; as such, the activity level of the female leopard geckos was higher by a factor of 1.08. With regard to the behavioural categories, the males showed a total of 7941 behavioural categories, and the females showed 10,096 behavioural categories, which is higher by a factor of 1.02. No significant difference between the females and males was observed for both behavioural units; the average number of behavioural elements per animal in the males = 1085, and the average number of behavioural elements per animal in the females = 1170 (*p*-value = 0.648), and the number of behavioural categories in males was 331, and in females, it was 337 (*p*-value = 0.843).

#### 3.2.2. Food Insects

Regarding the acceptance of the feed animals, only the intervention set can be taken into account, as feeding only took place during this data collection stage. The highest average number of behavioural units was realised when the geckos were fed with desert locust (*Schistocerca gregaria*) (19.95), followed by housefly (*Musca domestica*) (17.73) and house crickets (*Acheta domesticus*) (13.45). The same ranking was found concerning the behavioural categories: desert locust (*Schistocerca gregaria*) (4.61), followed by housefly (*Musca domestica*) (4.26) and house crickets (*Acheta domesticus*) (3.94). There was a significant change between the three different food insects for both behavioural units (*p*-value = 0.001) and behavioural categories (*p*-value = 0.003).

#### 3.2.3. Forceps Feeding

The shortest time span was 41 s, and the longest time span was 4 min and 2 s.

#### 3.2.4. Terrarium Size

There was no significant correlation between the terrarium size and activity or behavioural diversity (number of behavioural units) (*p*-value = 0.926) and diversity (*p*-value = 0.506).

#### 3.2.5. Body Temperature

There was no significant correlation between the activity level or behavioural diversity and body temperature (number of behavioural units, *p*-value = 0.322; diversity, *p*-value = 0.079).

#### 3.2.6. Qualitative Data

Leopard geckos are very well able to distinguish relevant stimuli from non-relevant stimuli, even at a distance to their enclosure. Since works in the literature about the behaviours of leopard geckos are very rare, three examples of our own observations from contexts other than the study were interesting. A terrarium with a front pane as wide as the table, inhabited by a couple of leopard geckos, stood for several years at a big kitchen table. Almost nothing that happened there, including the humans eating, talking, playing and listening to music, a big curious dog or a light in the evening, seemed to trigger a behavioural reaction from the geckos. Every evening, they came out of their hiding place and spent their time on a heat stone, their favourite resting place. But when a translucent box with crickets or locusts were put at the table, at a distance of about 80 cm from the front pane, the geckos became immediately active, staring at the insects and trying to go to them. In another situation in the same terrarium, at a distance of about 150 cm, another male leopard gecko walked over the table. Again, immediately, the male in the terrarium became very interested and active. In a third situation, three glass terrariums were placed side by side. In each terrarium, one male was housed. The panes were a little opaque due to limescale spots from spraying with water. The males were aware that their neighbours were males, too. On rare occasions, they showed the typical high position in order to threaten the others. Most of the time, they ignored each other, and sometimes they laid next to another, separated by the panes (all observations were made by the main author FK).

#### 3.2.7. Activity Time

Overall, there was a significant correlation between the activity level and time. The highest behavioural intensity was observed between 6 and 7 pm, roughly evenly decreasing with time. The individual data collection points show that the change in the behavioural intensity over time is significant for the intervention (*p*-value 0.001) and post-intervention (*p*-value = 0.001).

Which kind of behaviours are the most often realised ones, and which ones changed between the baseline, intervention and post-intervention?

The behavioural categories realised by the leopard geckos are presented in detail in the ethogram in Table 1 and Figure 10. For the purpose of analysis, it was useful to aggregate them to higher-ranking categories according to their behavioural meanings. There were four aggregate categories, which represent the majority of behavioural units: “sensory exploration” (33,114 observations), “walking around” (10,450 observations), “interest” (8598 observations), and “all resting behaviour” (5375 observations). The four main behavioural categories mentioned above all indicate well-being. As shown in Table 4, the quantity of behavioural units increased in three of four categories from the baseline to the intervention, and in two of the four categories, the quantity of behavioural units increased from the baseline to post-intervention. The “all resting behaviour” category showed a slight decrease from the baseline to the intervention and remained on a plateau. In order to further differentiate the meaning of this behavioural category, it was divided into two parts: “sense of security” (rest with eyes closed outside hiding place; rest outside hiding place; rest on elevated place) and “resting” (hiding place; rest under cover). The “sense of security” category shows a slight, non-significant decrease from the baseline to the intervention. Regarding the total change from the baseline to post-intervention, there was a significant increase in the number of observed behavioural units (*p*-value < 0.002). The “interest” category showed almost a doubling in the number of observations from the baseline to the intervention, and a further increase was observed from the intervention to the post-intervention. Compared to the baseline, the changes to the intervention and the post-intervention were significant (*p*-value < 0.020)

## 4. Discussion

The behaviours of a group of 18 adult leopard geckos of both sexes were recorded in their terrariums during their peak activity period between 6 pm and 11 pm in baseline, intervention and post-intervention data collection stages. The hypothesis to be tested was whether it is possible to increase the realisation of behavioural preferences by increasing the density of the stimuli through feeding the geckos with small insects, thereby increasing their well-being. The results showed that this type of enrichment led to a quantitative doubling in the activity levels from the baseline (total of 12,519 behavioural elements) to the intervention (total of 25,366 behavioural elements). And even 11 months after the introduction of small insect feeding (post-intervention total of 23,267 behavioural elements), the activity level was still significantly increased. The behavioural diversity, as the absolute number of behavioural categories for all 18 leopard geckos, also increased, although less than the behavioural intensity, between the baseline (5507 behavioural categories) and intervention (6451 behavioural categories) and between the baseline and post-intervention (6079 behavioural categories).

The focus of this study was the creation of evidence-based reptile husbandry regimes, cf., [5,12,19,43,55,85], to improve the living conditions and welfare of leopard geckos in both private and scientific contexts [8,43,86,87]. Welfare research on farm, laboratory and zoo animals is of increasing interest [1], but not private reptile husbandry [1,83]. The results showed that it was possible to sustainably activate leopard geckos in the terrarium by offering them behavioural opportunities through small insects in the form of acoustic, olfactory and visual stimuli. Such enrichment has already been shown several times to be an efficient means against boredom and a lack of stimulation and to increase well-being [22,24,30,56,87,91,92].

### 4.1. Differences Compared to Other Leopard Gecko Studies

All animals spent the entire study in their terrariums, which were much larger and more structured than what is usual in an ordinary scientific setup, e.g., [50,62,93,94], offering more hiding places, natural substrates, branches or roots and stones. This specific setup had four goals:

1. There is no justification for why animals used in science should be kept under poor husbandry conditions [6,24,92].
2. The quality and quantity of enclosures have significant impacts on the behaviours and welfare of the animals [87,88]. A change in the behavioural diversity or time budgets may be due to changes in the environment (e.g., a new terrarium) and need not have any impact on its welfare [31] but may nevertheless influence future behavioural expressions. It was shown, for example, in trouts (*Oncorhychus mykiss*), that enriched tanks promoted a better recovery from stress [95]. For corn snake (*Pantherophis guttatus*) and rat snake (*Pantherophis obsoletus*), it could be shown in different studies that a more structured terrarium leads to the animals having more interest in new objects, being better at problem solving and showing more explorative behaviour, greater behavioural intensity and well-being [41,42,43].
3. Since three data collection stages were conducted, and the whole study lasted one year, good housing conditions were necessary from a scientific point of view (true results) and also for ethical reasons (high degree of well-being of study animals), e.g., [6,33,55,79,82,96].
4. Small, minimalistic terrariums inherently limit the opportunities for action as they offer little choice to act out behavioural preferences and often act as severe stressors on the animals [9,22,26,33,55,81,91,93]. A study with laboratory rats (*Rattus norvegicus*) has shown that animals that were exposed to an increase in stimulus diversity through enrichment were better habituated and performed better in cognitive tests, had better spatial memory and object recognition and explored more. In other words, they were better off than the controls, and had a better welfare level. Furthermore, the release of some neurochemical parameters, like acetylcholine, was also reduced, which indicates reduced perceived distress [97]. A study with zebrafish [33] has shown that animals that were kept under typical laboratory conditions (small barren tanks) regularly exhibited abnormal behaviours, increased sensitivity to stress-inducing factors, lethargy, restriction of normal behavioural repertoire and other negative effects. Conversely, zebrafish that were kept under more complex and stimulating conditions were shown to have a higher rate of brain cell proliferation, faster learning or higher stress tolerance. Thus, animals in complex and stimulating environments provide more realistic, valid and valuable models for scientific interventions [98].

### 4.2. Resource and Animal Based Factors

Two complementary approaches are often proposed to assess well-being, e.g., [9,20,35,99]: resource-based aspects and animal-based aspects. Regarding the former, as described above, all geckos were in good health and were housed under better conditions than the norm in scientific setups, but not all geckos were housed under the perfect conditions of an ideal world. All leopard geckos had access to different gradations of brightness, humidity and warmth and thus always had choices to stay in a preferred place. The lighting duration more or less followed natural conditions, and the temperatures followed the seasonal cycle. The animals were allowed to hibernate for 3.5 months. Their diets consisted exclusively of insects, of which they received about 5 g/week/animal, as well as calcium vitamin supplementation. The health statuses of all participating animals were found to be good according to a reptile veterinarian. In cases where several leopard geckos were housed together, these animals had known each other and lived together for at least a year. No disharmony could be detected. Warwick et al. [100] matched a list of behavioural signs of quiescence and comfort as a tool to assess reptile welfare, including normal alertness, calmly smelling objects or air, subtle changes in body posture and orientation, unhurried body movements, a moderate to relaxed grasp on the handler and relaxed drinking, feeding, breathing and physical quiescence. All of these behavioural signs were regularly observed in the geckos that were part of the study. Some animals showed occasional signs of distress, which was analysed and discussed elsewhere [101]. A consideration of these resource-based factors showed that the animals were living in good housing conditions. There were no obvious reasons for pain, fear, suffering or permanent distress, and they had choices/control over diverse stimuli to express innate behaviours, which should have resulted in a reasonable degree of well-being [3,4,21,22,26,31].

However, this approach only covers part of the conditions that are necessary for an assessment of well-being. The other part is animal-based factors, or behaviour. Current evidence suggests that the evaluation of inputs valued by an animal via positive outcomes is the best method to assess positive welfare [9,55]. One key function of environmental enrichment is providing the animal with opportunities to express behaviour patterns for which it is highly motivated [19,22,24,29,30,55] and which are rewarding [19,30,102]. The conditions in a terrarium are often characterised by high predictability, a lack of stimuli and highly confined spatial conditions, which is the opposite of the conditions in the natural habitat in several ways. Accordingly, cognitive and behavioural skills are not or infrequently challenged, and the expression of behavioural preferences is limited by the restricted behavioural options [7,22,26,32,103], which cause boredom and compromise welfare.

As feeding with small insects satisfies a basic need, it does not wear out like other enrichments. In addition, the gecko necessarily has to move more to satisfy its appetite. As more individual insects/unit of weight are offered, the insects are distributed over a larger surface area and provide a higher encounter rate, which increases the stimulus density in the terrarium. The insects are perceived visually, olfactorily and acoustically, which further increases the stimulus diversity and probably also corresponds more to the conditions in the natural habitat. The geckos are motivated, physically and cognitively challenged and find it self-rewarding to respond to these stimuli [19,22,26,30,56,58], and it also initiates other behaviours in addition to foraging, for example, exploratory behaviour. Implementing such enrichment is also a preventive measure that helps to prevent atypical behaviour or unwellness. Providing a stimulus-rich environment is an essential method to create states of positive welfare [3,19,30]. For example, Maple et al. [87] reported about a successful target training with Mississippi Alligators (*Alligator mississipiensis*) in which two obese and lethargic Crocodylians were fed with a special diet to reward compliance. Feeding small insects that are free to roam can be understood as the antithesis of a widespread practice, forceps feeding, which a priori deprives the animal of a variety of behavioural opportunities, cf., [22,87]. During the preliminary study, the time it took for a leopard gecko to eat three adult house crickets (which is about the equivalent of 5 g of food) was measured. The time range was between 41 s and 4 min. Thus, it could be shown that this (common) feeding method reduces the time span in which a leopard gecko can act out its food-related behavioural preferences (hunting, eating and food-related exploration) to this extremely short weekly time span. In contrast, feeding with small free-roaming insects provides much more behavioural opportunities, which is associated with an increase in welfare. Similar results, but without a later evaluation, were shown in studies with fly river turtles (*Carettochelys insculpta*) in which these reptiles also spent significantly more time acquiring food through the presentation of appropriate behavioural opportunities than under standard conditions where food was simply thrown onto the water’s surface [47].

### 4.3. What Types of Behaviour Did the Leopard Geckos Display, and Can Indicators Regarding Their Well-Being Be Derived from This?

In the ethogram, 31 behavioural and 10 superordinate categories were differentiated. The higher-ranking category with the highest amount of behavioural intensity was “sensory exploration” (Figure 10), though this category had some methodological challenges. There were many clear observations of sensory perception, i.e., the gecko tilted its head and nostrils towards the ventilation grid as an expression of olfactory perceptions. In other cases, it turned its head following the movements of a desert locust, which could be interpreted as vision. Cases of tongue flicking indicated that the gecko had either perceived or was seeking olfactory stimuli [104,105,106]. But had the gecko also taken in olfactory stimuli while walking through the terrarium? If it moved its head a little bit and then stayed in that position for minutes, had it been looking the whole time? Had it continuously perceived its environment, in which nothing changed externally, with all its senses? And to what extent were these perceptions relevant to its well-being? Since these questions were not the subject of the present study, a conservative count of the behavioural elements was made, i.e., only the unambiguous elements (looking, smelling and tongue flicking) were recorded, from which it could be deduced that the figures significantly underestimate the actual extent of sensory perception. Chemical communication is a characteristic feature of different aspects of reptile biology [22,106,107], for example, prey detection and exploration. Tongue flicking serves as an important means to obtain chemical cues from the environment [104]. The vomeronasal organ is essential for detecting prey in many squamates [108].

The behavioural element of “position alteration” was also added to the superordinate “sensory exploration” category. This expresses small body movements, i.e., less than one step with one foot. The majority were head movements. It seems plausible that every head movement was connected with a sensory perception, e.g., looking. In other words, this category summarises all situations of sensory perception that cannot be differentiated into a single one. Here, too, it can be assumed that the actual number of sensory perceptions is underestimated. Furthermore, it seems plausible that a few head movements were also not primarily associated with a perception. Due to the above-mentioned problems, no simultaneously occurring sensory perceptions were recorded, and the recording of acoustic perceptions was omitted, although the latter clearly plays a role in the localisation of prey by leopard geckos (F. Krönke unpublished data).

The outstanding frequency of behavioural elements from this super category and their significant increase between the baseline and the intervention or post-intervention indicate that sensory exploration represents a central behavioural preference for leopard geckos under terrarium conditions. This conclusion is consistent with the approach of “asking animals what they want” [27,31,54,92,108,109]. It could thus also be stated that a wealth of sensory perception in leopard geckos, without forms of atypical behaviour, a state of good health, high-level housing and husbandry and the absence of lasting distress might be an indicator of well-being as it is a self-rewarding behaviour and therefore evokes a positive mental state, which is closely associated with good welfare and correlated with the leopard geckos’ health [4,9,13,19,25,27,30,35,110,111].

The second most frequent category was “walking around” (Figure 10). This category summarised different kinds of larger movements (at least one snout to vent length). It played a role in exploratory behaviour but appeared to be less important than sensory exploration. There was a significant increase from the baseline to the intervention and also from the intervention to post-intervention. It is a space-using behaviour.

The third overarching category was “interest” (Figure 10). Observations in this category almost doubled from the baseline to intervention and further increased from the intervention to post-intervention. This category includes the “high position”, where at least the front legs, and sometimes also the hind legs, are stretched. This was neither the classic fright reaction, nor was it sexually motivated. The second type of behaviour in this category was “head up”, where the head is stretched upwards at an angle of 45–90°. Sometimes, this position was adopted when, for example, flies were running along the deck. As this position also elevates the eyes compared to the normal head position, it is likely that the gecko is increasing its field of vision and gaining additional olfactory stimuli. In such situations, attention or interest appeared to be indicated. In other situations, no optic stimuli were visible, and the gecko remained in this position for minutes, sometimes with its eyes closed. Another behaviour of this group is a “change of body orientation”, i.e., at least one step with one leg and less than one snout-to-vent length of locomotion. Mostly the position of the head and the orientation of the body axis were changed. The behaviour of this category was not primarily motivated by sensory perception, nor was its purpose locomotion. Changes in the body orientation often followed position alterations, i.e., very small movements of the head. This behaviour seems most plausible as a kind of attention behaviour. After a sensory perception, the motivation to (re)act was present, but not strong enough as to be followed by an action, or the situation was evaluated in such a way that a reaction did not seem necessary. This could be an expression of differentiated behaviour, like adapting attention and behaviour to (new) environmental stimuli, as described by Szabo et al. [112,113].

The fourth category was “resting behaviour”. The resting levels of all geckos dropped non-significantly from the baseline to the intervention, as well as from the intervention to the post-intervention, and dropped significantly from the baseline to the post-intervention. The results primarily indicate two things: the variation between the data sets is low, leading to the explanation that the resting behaviour is an intrinsic component of the normal behaviour of leopard geckos. Furthermore, it can be concluded that a stimulus-poor environment (baseline conditions) does not necessarily generate lethargy at the population level. Periods of sleep and rest are indicative of good welfare [9]. Furthermore, due to their ectothermic metabolisms, reptiles are often less active than endotherms [7,114], which means frequent and long resting periods are part of normal behaviour in many species. Since the time budget for rest was about the same in all three data sets, it might be seen as an indication of a certain degree of welfare even before the intervention began.

To further differentiate the meaning of resting behaviour, these behaviours were divided into two categories: a “sense of security” (rest with eyes closed outside hiding place; rest outside hiding place; rest in an elevated place) and “resting” (hiding place; rest under cover) (Figure 10). While the second category (“resting”) was characterised by the head and/or body being under a cover, which might relate to a need for security, the first category might express a feeling of security, which is why the second category is called “sense of security”. The head and/or body were mostly out of cover and the eyes were often closed, implying an awareness of the extent and structure of the terrarium, daily routines and that no acute threats were assumed. Leopard geckos can afford to be vulnerable. This assumption is aligned with that of Mohanty et al. [115], who states that the sleeping behaviour in reptiles is always closely connected to predator avoidance and with the qualitative observation that leopard geckos are capable of assessing or evaluating their environment, including the immediate surroundings outside the terrarium, very accurately. Regarding the total change from the baseline to the post-intervention and from the intervention to the post-intervention, a significant increase in the number of behavioural units was observed. These changes illustrated that behaviour associated with perceived security increased along with activity. As such, it might be seen as a second line of evidence that feeding enrichment improves the welfare of leopard geckos, cf., [13,35,110]. Therefore, there is a case for conceptualising the sense of security behaviours as an indicator of feelings of safety and thus well-being.

In summary, the increase in the behavioural intensity as a result of the increase in the stimulus density through small insect feeding suggests that under baseline conditions, leopard geckos lacked the opportunity to engage in appetitive behaviours and the realisation of apparent goals, which was associated with a reduction in well-being. However, it is difficult to make a clear statement on how large the measurable effect (e.g., increase in behavioural intensity) must be in order to speak of a substantial improvement in the situation, and how large the range of a behavioural change may/should/must be or how a target value can/should be defined. The classical answer is to use behavioural data from the natural habitat as a basis for comparison, e.g., [28,29,116]. However, as the design of the natural habitat, the behavioural profile, population-related or individual differences and many other factors differ significantly between the natural habitat and captivity, it is questionable to what extent the behavioural data or time budgets from the natural habitat can be transferred in a 1:1 ratio.

### 4.4. Other Correlations

#### 4.4.1. Housing

For reasons of practical necessity, the 18 leopard geckos were kept in seven different terrariums and in varying group sizes. With regard to the above-mentioned behavioural categories, no statistically significant correlation with the terrarium size could be demonstrated. Leopard geckos are very adaptable in terms of their living conditions in a terrarium. Part of the objective of the study was to answer the question of how the selected leopard geckos behave and feel under the given circumstances, i.e., within their terrarium, which has been familiar to them for several months, and whether they change their behaviours within this environment. This is a common approach in zoo animal research, where it is implicitly or explicitly assumed that husbandry conditions in different zoos are never identical and that the data are nevertheless comparable, e.g., [3,4,35,88,116,117,118].

The group housing of leopard geckos in the study was driven by the aim to create more stimulus-rich husbandry conditions. Keeping males separate is the consequence of the fact that male leopard geckos do not tolerate each other in ordinary small terrariums and will attack and injure each other. However, this is the second best option from a welfare perspective, cf., [119]. Providing enrichment, as in this study, was effective, as the husbandry system did not have an effect on the increased activity and behavioural variability and thus improved welfare. There was no statistical significant difference in behaviour between the male and female animals. And likewise, no significant correlation could be found between the body temperature and behavioural intensity or behavioural diversity.

#### 4.4.2. Behavioural Diversity and Well-Being

According to the natural living concept, which states that an animal that is able to perform natural behaviours is in a state of positive welfare [31,120], high behavioural diversity is thought to be an indicator for well-being and, vice versa, low diversity is indicative of poor well-being and lethargy, though there are differing views about behavioural diversity as well. Animals in captivity always have a restricted behavioural repertoire compared to wild counterparts; therefore, the question also arises as to whether behavioural diversity is an absolute or a relative measure for animals under captive conditions [20,28,35,103,116,121]. This may be particularly true for ectotherms, who show a restricted diversity because they have everything they want, do not need to search or explore and may instead be motivated to save energy. They may experience a high degree of well-being yet exhibit few but positively valenced behaviours that may indicate welfare [25,27,31,122].

In these data sets, there was a significant increase in the behavioural diversity (behavioural categories) from the baseline to the intervention, but there was no significant increase between the baseline and post-intervention. Since feeding insects were offered to the leopard geckos during the intervention but not in the post-intervention, it seemed obvious that the increase in the behavioural categories was due to actual stimuli. From this—according to theory—it might be deduced that well-being decreased in the post-intervention data set. This interpretation might not be correct for several reasons:

1. Even without actual stimuli, the increase in the behavioural intensity (behavioural units) was still significant 11 months after the enrichment, exhibiting twice the level of activity compared to the baseline.
2. As cited in Materials and Methods, there were neither behavioural signs of reduced well-being nor any changes made to the high housing and husbandry quality.
3. As highlighted by Burghardt [7] and Warwick et al. [92], due to their ectothermic metabolisms, the activity levels of reptiles are different to those of mammals or birds [114]; however, the model of broad behavioural diversity as an indicator of good welfare was built on research on endotherms [5,6,7,8].
4. The long observation time of this study indicates the high reliability of the data.

It might be assumed that the leopard geckos of this study, who realised an average of 19.7 behavioural categories out of 31 possible categories at the baseline (see Table 3), already showed an acceptable degree of well-being, even at the baseline. It therefore seems plausible that an additional increase in the behavioural diversity was not a necessary condition for an increase in well-being, cf., [110]. This is in line with some authors who do not exclusively understand behavioural diversity as the number of different behavioural categories, but also as the frequency of behaviour, e.g., [120], and that behavioural diversity without context has only limited significance [27]. The context is that the behavioural intensity increased significantly between the baseline and intervention as well as between the baseline and post-intervention. This is, as discussed above, a strong indication that the leopard geckos felt well because they significantly increased the frequency of behaviours that they were highly motivated to perform, which were self-rewarding, and at the same time, they did not show any behaviours that indicated discomfort, even if their behavioural diversity was less than expected. It therefore might be worthwhile to conduct further studies with other reptile species to test the diversity model.

### 4.5. Was It Trivial to Conduct This Study?

Critics might say yes, because it is a compelling necessity for a leopard gecko to move more for its food when it is smaller to obtain the same amount of food. However, the results clearly showed that only a minimal proportion of the activity budget was spent on foraging, and that even after 11 months, the total activity was still about twice as high as before, showing that conducting this study was not trivial. Binding et al. [1] demonstrated in a meta-analysis that enrichment is one of the most effective strategies for promoting psychological well-being in animals, and how an animal feels is seen as what counts the most [55,99]. The longitudinal nature of this study with multiple timelines is a substantial advantage.

The intervention survey was able to show that an increase in the environmental stimulus density was successfully established in the leopard geckos by introducing small insects as active, free-roaming prey. Many studies end at this point and only show that the respective animal responded to the stimulus [22], e.g., [50,51,123,124]. What is essential to sustainable enrichment is the achievement of the goal, i.e., the promotion of well-being through the creation of specific behavioural opportunities [22,87].

## 5. Conclusions

“In using animals for our purposes we exercise varying degrees of control over the quality and duration of their lives. That control gives us the opportunity to manage them humanely. Moreover, using them for our own purposes, not theirs, requires us to do so. Accordingly, we have an ethical ‘duty of care’ towards the animal in our control and this translates into a practical obligation to keep their welfare [...]” [55] at high levels. And in the same sense, Boissy et al. [25] states the following: “If we want to maximize welfare, then the aim must be to give the animals as good quality of life as possible all the time”. Whitham et al. [13] urge that all zoo animals should be offered challenges that induce positive affective states throughout life, and not only when abnormal behaviours occur. It should be added that this premise should apply equally to all privately kept reptiles.

The leopard gecko is probably the most widely distributed pet lizard in private husbandry [60] and has become a model for some types of research questions, even in science, e.g., [64,67,68]. This is due to its characteristic as an ecological generalist, because it is easy to keep and breed and it is considered a good “starter reptile” for beginners as it “condones” husbandry mistakes, even for extended periods. However, being a mass species is not a second-class classification. They, too, have an equal claim to good well-being as all animals in human care. “As many now recognise, environmental ‘enrichment’ is not an extra benefit that we may choose to provide as a luxury if the time budget permits; it may be essential for proper management, even for reptiles!” [7].

Feeding enrichment with small insects is a step in this direction that can be realised by any leopard gecko keeper without extra effort or higher costs. The triumphant success of the internet has resulted in both a myriad of information on reptile husbandry and an enormous increase in the accessibility of most species. Unfortunately, the past decade has also seen the spread of a trend towards a medium to low husbandry quality in reptiles (cf., various leopard gecko internet platforms). This makes it obvious that the quality of husbandry is not only related to the available information, but also to the attitude of the reptile owner [37]. During the decades of the emergence of terraristics, e.g., in Germany more than 140 years ago, the prevailing idea was to provide the animals in the terrariums with a “copy” of their natural habitat to ensure their well-being, e.g., [125,126,127,128,129]. For those who feel responsible for their reptiles, this approach still applies [9,90,92]. Unfortunately, a “convenience minimalism” has spread today that follows certain maxims: spend as little money as possible; do not leave home when home delivery is an option; do not buy books because the internet is free; and do not make commitments—sell it when you become bored.

We hope to have shown that it is possible to increase the well-being of leopard geckos with minimal effort.

## Figures and Tables

**Table 1:** Ethogram of the leopard gecko.

Ethogram of the Leopard Gecko			
	Abbreviation	Behavioural Categories	Definition
resting behaviour I			
1.	hp	hiding place	resting within a hiding place, not active, complete body or for at least 2/3 of it not visible, head always hidden, no observation of the outside possible
2.	ruc	rest under cover	animal is partly visible, cover is open at least at one side, observation of the outside is possible
sense of security			
3.	ro	rest outside	resting outside any hiding place or cover, not physically active, lasting at least 3 s, not the ordinary break between all behavioural units
4.	rep	rest elevated place	rest most of the time outside cover at elevated structures like stones, roots, cork tubes
5.	rec	rest eyes closed	resting with or without cover, with one or both eyes closed, indicating a sense of security
walking around (large movements)			
6.	wa	walk around	walk of at least one body length, mostly in connection with explorations
7.	wasm	walk around slow motion	walking with a strongly reduced speed, mostly in context of prey capture, exploration or social contacts
8.	clim	climbing	explorative or targeted action, both directions: up and down on a stone, root, etc.
sensory exploration			
9.	pa	position alteration	mostly isolated head movements but also position movements of the body without going a step, includes sensory perceptions like smelling or looking, which are sometimes difficult to detect
10.	look	looking	sensory perception/exploration during any activity; head often follows a stimulus
11.	sme	smelling	sensory perception/exploration during any activity; head often follows a stimulus and nose touches or becomes very close to the object of interest
12.	tf	tongue flicking	consolidated category sensory perception/exploration during any activity, head often follows a stimulus and tongue touches the object of interest; also, after drinking or eating, sometimes tongue flicks into the air, and sometimes the mouth is opened and the tongue does not transcend the jawbone
interest			
13.	coo	change of body orientation	change of body orientation, with at least one leg moved, and body moves less than a whole body length, often a realignment of the body axis
14.	rhp	rest in high position	at least the forelegs, and in some situations, also the hind legs, are strung out, often in combination with hu; if all 4 legs are strung out, it is not the classic fright reaction, which is directed to a threat it could last a few seconds to several minutes, probably an expression of interest and sensory perception/exploration directed toward a stimulus; sometimes with closed eyes
15.	hu	head up	head is directed upward between 45 and 90°, often together with rhp, probably sensory perception/exploration directed toward a stimulus, e.g., tearflys walking on the ceiling of the terrarium
foraging behaviour			
16.	ttv	tail (tip) vibrations	all types of tail movements, mostly in context with prey or social contact
17.	snap	snap	snap a prey, not a synonym with eat, because a snap can be unsuccessful
18.	bj	bag jump	jump towards a prey in order to bag it
19.	eat	eating	eat a prey
basic needs			
20.	drink	drinking	drink water
21.	def	defecate	defecate
22.	gape	gape	single wide opening of mouth, mostly at the beginning of activity period
23.	cl	cloaca licking	cloaca licking
ambivalent behaviour			
24.	dig	digging	mostly near the front pane; single movements to construction of holes that can harbour the gecko
25.	pw	pane walking	walking along the front pane; in some situations, this is a sign of low well-being
26.	lop	look out pane	mostly front pane, probably an expression of interest and sensory perception/exploration, often together with pa, rhp, pw
Indication of distressed behaviour			
27.	ps	pane scratching	scratching at front pane or ventilation grid; clear sign of low well-being
28.	vps	vertical pane standing	standing on hind legs at front pane, often together with scratching movements of forelegs, clear sign of low well-being, motivation to escape
29.	rhp fp	resp high position at front pane	head is directed toward front pane or in angle of 90° to fp; in some situations, this is a sign of sensory perception/exploration, and in others, it is a sign of low well-being or motivation to escape if ps or vps are also shown in a temporal context
indication of low well-being			
30.	mpr	mouth at pane rubbing	often in combination with tf, sme, pa and coo, very short duration, rare, sensory perception/exploration, or sign of low well-being, with motivation to escape
31.	wmp	wriggling movements at pane	always in combination with coo or pa, short in duration, rare, clear, discrete repetitive behaviour and sign of low well-being, acute stress and motivation to escape, exclusively in connection with front pane
resting behaviour II			
		resting, no behaviour (not counted)	little breaks (less than three seconds) in a behavioural sequence, respectively, the “stops“ between single behavioural elements

**Table 2:** Husbandry type.

ID	Sex	Housing	Usable Space cm^2^
1	m	single	2500
2	m	single	2500
3	m	single	2500
4, 5	f, f	pair	5000
6	m	single	2500
7	m	single	4000
8, 9	f, f	pair	3200
10	m	single	3300
11, 12	f, m	pair	3300
13, 14	f, m	pair	7000
15, 16, 17, 18	f, f, f, f	quartet	21,000

m = male, f = female.

**Table 3:** Significance level of behavioural diversity (behavioural categories) referring to 31 possible behaviours (ethogram) and data sets.

*p*-Values	Baseline_1_2	Intervention_1_3	Post-Intervention_2_3
behavioural diversity	0.070	0.116	0.005
	**Baseline 1**	**Intervention 2**	**Post-intervention 3**
**average behaviours performed**	19.7	21.4	18.5
range of variation	11	12	11
minimum	14	15	14
maximum	25	27	25
median	19.5	21	18

Baseline = data set 1. Intervention = data set 2.

**Table 4:** Changes in behavioural quantity.

Table: Changes in Behavioural Quantity							
Data Set	Sensory Exploration		Walking Around		Interest		All Resting Behaviour
		Factor		Factor		Factor	
baseline	5723	-	2431	-	1936	-	1848
intervention	13,477	2.35	5696	2.34	2914	1.51	1734
post-intervention	13,914	2.43	2323	0.96	3748	1.94	1793

Factors are related to baseline data set.

## Data Availability

No new data were created or analyzed in this study. Data sharing is not applicable to this article.
